# Supplementary material for: The Antimicrobial Potential of the Hop (Humulus lupulus L.) Extract against Staphylococcus aureus and Oral Streptococci
Source: Pharmaceuticals (Basel). 2024 Jan 27;17(2):162. doi: 10.3390/ph17020162 (PMC10893079; doi:10.3390/ph17020162)
Supplement: Supplementary file 1 [file pharmaceuticals-17-00162-s001.zip › pharmaceuticals-2789634-supplementary.pdf]

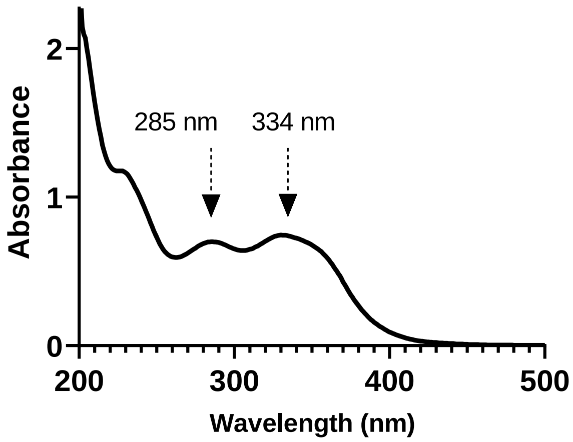

Figure S1. UV-Vis spectra of the working solution of the final extract at a concentration of 40 mg/mL after purification. The absorbance (cm^{-c}) has been measured in the wavelength range of 200–500 nm. Arrows show absorbance peaks at λ = 285 nm and λ = 334 nm that correspond to the characteristic absorption lines of APs.

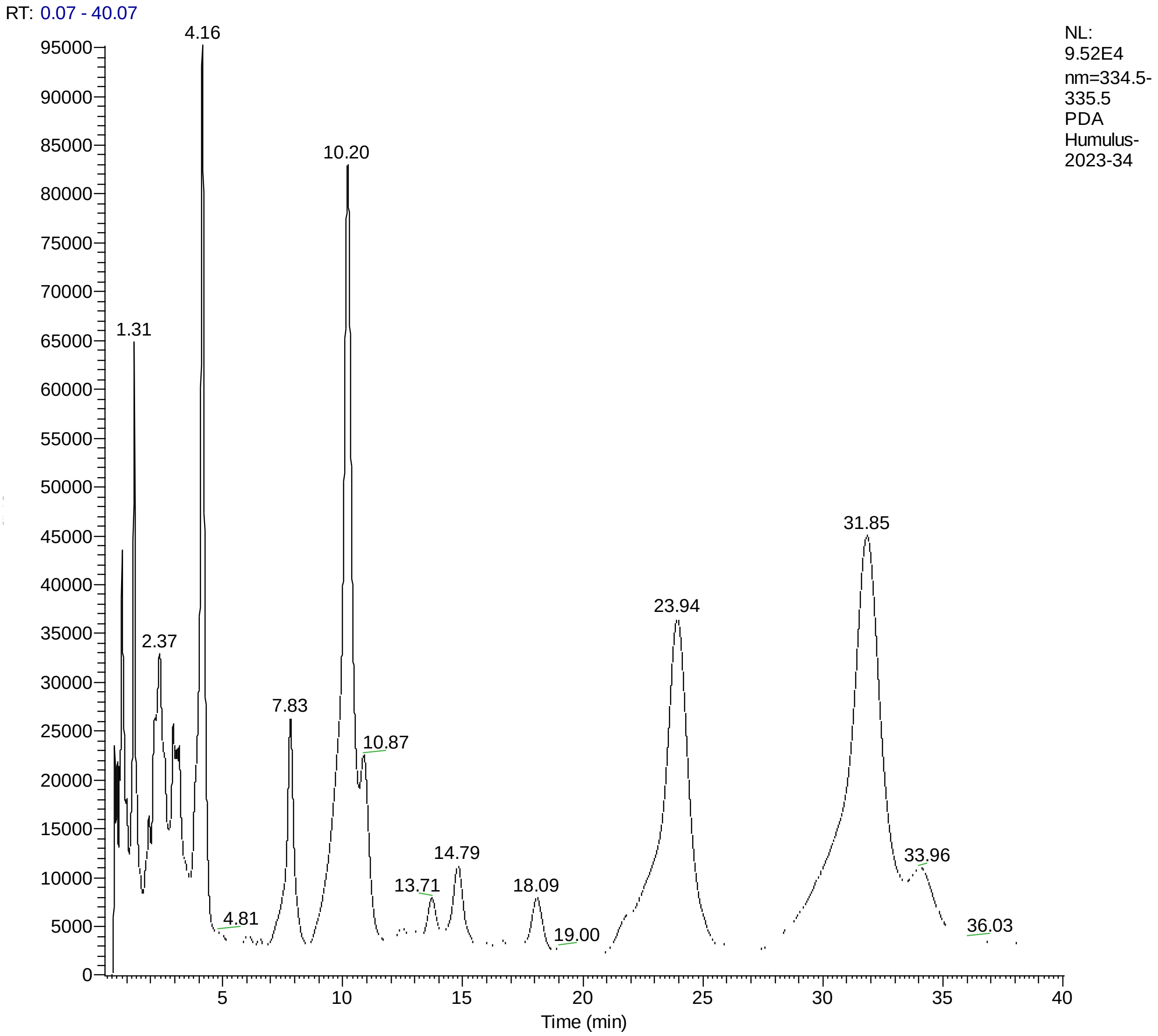

Figure S2. The typical LC-MS chromatogram of the extract *H. lupulus.* The LC-MS performed on Thermo finnigan LCQ fleet equipped with BDS hypersil C 18 2×150 mm column. The chromatography has been performed at isocratic flow at 0,8 ml/min in a solvent system of acetonitrile-water (10% formic acid and 0.01% i-propanol) in a ratio of 1:1 (v/v) and detection on a PDA detector at 335 nm.

Table S1. The chemical structures of the main active compounds (2',4',6',4-tetrahydroxy-3'-geranylchalcone and acylphloroglucides) identified in the hop extract by LC-MS

1	Humulone	$C_{21}H_{30}O_5$	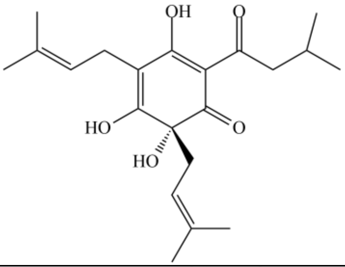
2	Cohumulone	$C_{20}H_{28}O_5$	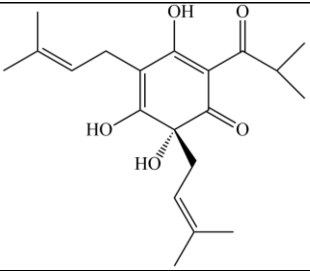
3	Lupulone	$C_{26}H_{38}O_4$	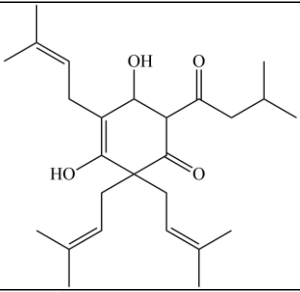
4	Colupulone	$C_{26}H_{38}O_4$	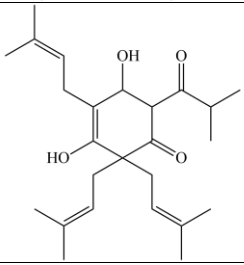
5	2',4',6',4-tetrahydroxy-3'-geranylchalcone	$C_{25}H_{28}O_5$	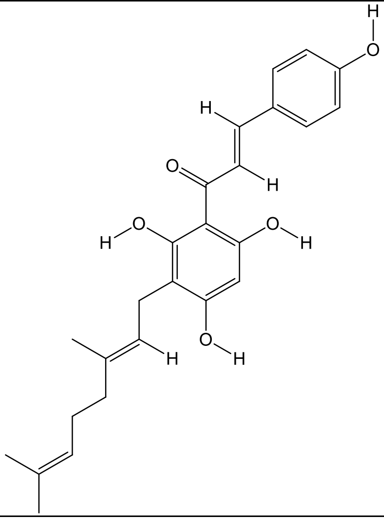
